# Health-related quality of life among adults with and without hypertension: A population-based survey using EQ-5D in Shandong, China

**DOI:** 10.1038/s41598-017-15083-4

**Published:** 2017-11-02

**Authors:** Li Zhang, Xiaolei Guo, Jiyu Zhang, Xi Chen, Chengchao Zhou, Dandan Ge, Yangyang Qian

**Affiliations:** 1 Department of Social Medicine and Health Service Management, School of Public Health, Shandong University, Jinan, China; 2 Shandong Center for Disease Control and Prevention, Jinan, China; 3 Collaborative Innovation Center of Social Risks Governance in Health; School of Public Health, Shandong University, Jinan, China

## Abstract

Previous studies have focused on health-related quality of life (HRQoL) in hypertensive individuals, but few studies have attempted to compare HRQoL between hypertensive and normotensive individuals using the EQ-5D in China. Based on a survey of 3509 adults aged 18 + years, we compared HRQoL between hypertensive and normotensive individuals using a chi-square test, t-test and multi-linear regression model. The results indicated that HRQoL in hypertensive individuals was poorer than that of normotensive individuals in all domains of the EQ-5D and its utility index. In addition, education, complications, household income, and family history of HBP were associated with HRQoL among the hypertensive patients. Factors including age, education, household income, health expenditure, place of residence, and family history of high blood pressure (HBP) were found to be associated with HRQoL in normotensive individuals. Interventions targeting at-risk subgroups, such as modifying existing health insurance schemes to improve them for poor individuals, might be helpful to improve HRQoL.

## Introduction

As a result of the accelerated process of population ageing, rapid urbanization and worldwide lifestyle changes, an epidemic of hypertension is emerging globally^1^. Hypertension is most commonly a non-infectious, chronic disease, and it is recognized as a major risk factor for cardiovascular diseases, other chronic diseases (e.g., diabetes, renal disease) and overall mortality, which affects all segments of the population^2,3^. The burden of hypertension continues to increase. In the first 25 years of the 21^st^ century, the burden of hypertension is expected to increase by 60% globally^4,5^. Hypertension also causes a high burden in China. The disability-adjusted life years (DALY) due to hypertension account for 12% of the total DALY, and approximately 2 million premature deaths result from elevated blood pressure every year in China^2^.

Health-related quality of life (HRQoL) is the health status (including physical, emotional and social aspects) reported by individuals themselves^6^. HRQoL assesses how an individual’s well-being may be affected by a disease, disability, or disorder over time^6,7^. It is usually measured using questionnaires. In the past few decades, hundreds of HRQoL questionnaires have been developed to assess the quality of life of patients with various illnesses^8–10^. Such questionnaires can be generalized into two categories: specific instruments (e.g., LC-13, HADS) and generic instruments (e.g., SF-36). Many of the currently existing condition-specific instruments are not adequate, and generic measures lack simplicity. The EQ-5D, a generic measure of HRQoL developed by the EuroQol Group in 1990, can address these issues and effectively evaluate HRQoL^11^.

Numerous studies have addressed the impact of hypertension on HRQoL^12,13^. Carvalho MV *et al*. used SF-36 to evaluate the HRQoL of hypertensive versus non-hypertensive individuals in Brazil and found that hypertension impaired the HRQoL of patients who suffered from it^14^. Bardage C *et al*. found that persons with hypertension had lower HRQoL than normotensive individuals in Sweden^15^. A population-based study in China found that persons with hypertension experienced lower HRQoL compared with those without hypertension^16^. However, most previous studies measured HRQoL using rather long generic instruments, such as SF-36, WHOQOL-100, or WHOQOL-BREF. Further, in many studies, only hypertensive patients were included, and it was not clear who served as the reference group. To date, very few studies concerning the HRQoL of hypertensive patients in China have evaluated HRQoL using the EQ-5D^17,18^. There is also a lack of research to compare HRQoL between hypertensive and normotensive individuals in China.

The present study’s overall goal is to empirically compare HRQoL between hypertensive and normotensive individuals in China using the EQ-5D. This study has two specific objectives. First, we compare HRQoL, its domains and utility index between hypertensive patients and normotensive individuals. Second, we identify risk factors for HRQoL in hypertensive and normotensive individuals.

## Methods

### Study population and data collection

The present study uses the interim survey data of the Salt Reduction for Preventing Hypertension Project (SRPHP) in Shandong province. The SRPHP was organized by the Government of Shandong Province and the National Health and Family Planning Commission of the People’s Republic of China and was conducted by Shandong Center for Disease Control and Prevention. This interim survey was conducted in Shandong province, China, in 2014.

The baseline survey of this project was conducted in 2011. Eight urban districts and twelve rural counties were randomly selected as the study sites based on the GDP per capita and location. Two sub-districts or townships were selected in each sampling site. Likewise, three communities (or villages) were selected in each sub-district (or township). Finally, 100 residents (over 18 years) were randomly selected from each community or village. In the interim survey, two of the eight urban districts (Fushan District, Laicheng District) and four of the twelve rural counties (Gaomi, Gaotang, Junan, and Zoucheng) were selected as study sites based on the GDP per capita, location of the sites (eastern, central and western), and proportion of urban and rural population in Shandong (1:2). Two hundred residents (over 18 years old) were randomly selected from each community or village. In total, 3509 eligible participants were included in this study.

### Data collection

A standardized questionnaire was designed to collect data, including socio-demographic information on the individuals and households, health-related behaviours, health conditions (hypertension and its complications, family history, and the economic burden of hypertension), quality of life, and utilization of health services. All participants were interviewed face to face in an isolated room in their houses by trained postgraduate students from the Shandong University School of Public Health and staff from the Shandong Centre for Disease Prevention and Control. The interviewers were trained in the background of the project and investigation skills. To ensure quality, completed questionnaires were carefully checked by quality supervisors after the interviews each day.

### Dependent Variables

HRQoL was measured by the EQ-5D. The EQ-5D enables participants to classify their health condition in five dimensions, and its reliability and validity have been demonstrated as acceptable in China^19^. These domains define health status in terms of mobility, self-care, usual activities, pain/discomfort, and anxiety/depression^11^. Each domain is divided into three levels (no problems, some problems and extreme problems)^8,10^. The EQ-5D index score is generated by applying societal preference weights to each of the five dimensions according to the Chinese population-based time trade-off (TTO) model^19^. Higher scores indicate better HRQoL.

### Independent variables

All hypertensive patients were identified by the question, “Have you been diagnosed with hypertension by a doctor?” If yes, respondents were also asked if they had been diagnosed with any of the following complications: angina pectoris, myocardial infarction, stroke, or hypertensive nephropathy. Complication was defined as having any of the diseases listed above.

Socio-demographic factors included age (<40, 40+, 50+), sex (male, female), education (primary or below, junior, and senior or above), occupation (farmers and others), residence (rural, urban), household income (<20000, 20000+, and 40000 + RMB Yuan), health insurance (yes, no), and health expenditure (<900, 900+, and 1800 + RMB Yuan).

Health behaviour-related factors included sleeping time and drinking (yes, no). Sleeping time was recorded as <7, 7+ and 8+ hours per day.

Hypertension-related information included family history of high blood pressure (HBP) (yes, no), complications (yes, no), anti-hypertension medication in the past two weeks (yes, no), willingness to acquire health-related knowledge, and HBP knowledge level. Willingness to acquire health-related knowledge was coded as “yes” or “no” by asking the question, “Do you take the initiative to acquire health-related knowledge?” Hypertension knowledge level was measured by three questions: “Do you know the association between diet and obesity with hypertension?”, “Do you know the association between overweight and hypertension?”, and “Do you know the association between smoking and hypertension?” Each question was coded as yes (1) or no (0). Higher scores indicate better hypertension knowledge level.

### Data analysis

We used SPSS 18.0 to analyse the data. When analysing the data, we weighted the sample by stratification and clustering to adjust for the sampling design. The socio-demographic characteristics of individuals with and without hypertension were compared by chi-square for categorical variables. A t-test was employed to compare the five domains of the EQ-5D and the EQ-5D index between hypertensive and normotensive individuals. Multiple stepwise linear regression analyses were used to identify associated factors of EQ-5D among hypertensive and normotensive individuals, respectively. The statistical significance in this paper was set at the 5% level.

### Data sharing

No additional data available.

#### Ethical consideration

This study was approved by the Ethical Committee of the Shandong Center for Disease Control and Prevention. The investigation was conducted in accordance with the principles of Declaration of Helsinki and approved guidelines, and it was carried out after the informed consent of all participants was obtained.

## Results

Table 1 shows the basic information about the participants. Of the 3509 respondents, 419 participants were hypertensive patients. Generally, the majority of the participants had the following characteristics: female (52.4%), aged 50 and above (39.8%), a junior or below education level (82.6%), farmers (63.3%), non-drinkers (73.3%), rural residents (87.7%), no history of high blood pressure (79.1%), covered by health insurance (77.4%), and willing to acquire health-related knowledge (62.2%). Among the hypertensive patients, 52.7% had no complications, and 72.3% had used antihypertensive medication in the last two weeks.

**Table 1:** Comparison of baseline characteristics between hypertensive and normotensive individuals in Shandong, China, 2014

Variables	N (%)	Hypertensive	Normotensive (%)	χ^2^	P
Observations	3509	419 (12.6)	3090 (87.4)		
Gender				0.347	>0.05
Male	1672 (47.6)	194 (46.3)	1478 (47.8)		
Female	1837 (52.4)	225 (63.7)	1610 (52.2)		
Age				292.195	**<0.001**
<40	1187 (33.8)	18 (4.3)	1169 (37.8)		
40-	927 (26.4)	81 (19.3)	846 (27.4)		
50-	1395 (39.8)	320 (76.4)	1075 (34.8)		
Education				74.007	**<0.001**
Primary or below	1407 (40.1)	248 (59.5)	1159 (37.6)		
Junior	1491 (42.5)	127 (30.5)	1364 (44.3)		
Senior or above	601 (17.1)	42 (10.1)	559 (18.1)		
Occupation				14.948	**<0.001**
Farmers	2221 (63.3)	301 (71.8)	1920 (62.1)		
Others	1288 (36.7)	118 (28.2)	1170 (37.9)		
Household income (RMB Yuan)				104.443	**<0.001**
<20000	1191 (33.9)	234 (55.8)	957 (31.0)		
20000-	1208 (34.4)	109 (26.0)	1099 (35.6)		
40000-	1110 (31.6)	76 (18.1)	1034 (33.5)		
Sleeping time (hours per day)				22.729	**<0.001**
<7	1251 (35.7)	187 (44.6)	1064 (34.4)		
7-	1652 (47.1)	153 (36.5)	1499 (48.5)		
8-	606 (17.3)	79 (18.9)	527 (17.1)		
Health expenditure (RMB Yuan)				26.697	**<0.001**
<900	1336 (38.1)	122 (29.1)	1214 (39.3)		
900-	916 (26.1)	101 (24.1)	815 (26.4)		
1800-	1257 (35.8)	196 (46.8)	1061 (34.3)		
Drinking				0.209	>0.05
No	2572 (73.3)	311 (74.2)	2261 (87.9)		
Yes	937 (26.7)	108 (25.8)	829 (12.1)		
Residence				4.519	**<0.05**
Urban	432 (12.3)	65 (15.5)	367 (11.9)		
Rural	3077 (87.7)	354 (84.5)	2723 (88.1)		
Family history of HBP^a^				177.835	**<0.001**
No	2774 (79.1)	227 (54.2)	2547 (82.4)		
Yes	735 (20.9)	192 (45.8)	543 (17.6)		
Complications				—	—
No	—	198 (47.3)	—		
Yes	—	221 (52.7)	—		
Antihypertensive medication^b^					
No	—	116 (27.7)	—		
Yes	—	303 (72.3)	—		
Health insurance				2.416	>0.05
No	791 (22.5)	82 (19.6)	709 (23.0)		
Yes	2717 (77.4)	337 (80.4)	2380 (77.0)		
Willingness to acquire health-related knowledge				13.163	**<0.001**
No	1325 (37.8)	191 (45.7)	1133 (36.7)		
Yes	2184 (62.2)	227 (54.3)	1957 (63.3)		
HBP knowledge level				8.521	**<0.05**
0	571 (16.3)	88 (21.0)	483 (15.6)		
1	293 (8.3)	37 (8.8)	256 (8.3)		
2	518 (14.8)	60 (14.3)	458 (14.8)		
3	2127 (60.6)	234 (55.8)	1893 (61.3)		

^a^HBP: high blood pressure.

^b^refers to antihypertensive medication in the past two weeks.

The individuals with hypertension experienced lower scores in all domains of the EQ-5D and the EQ-5D index compared with those without hypertension (Table 2), and the differences were statistically significant in all domains of the EQ-5D and the EQ-5D index (P < 0.001) (Table 2).

**Table 2:** Mean scores of different domains of the EQ-5D in populations with and without hypertension in Shandong, China, 2014

Domains of EQ-5D	Hypertensives	Normotensives	Difference	P
	(M ± SD)	(M ± SD)	(95%CI)	
N	419	3090		
Mobility	93.18 (18.20)	98.64 (8.43)	−5.46 (−7.23, 3.68)	**<0.001**
Self-care	96.40 (15.47)	99.14 (7.10)	−2.74 (−4.25, 1.23)	**<0.001**
Usual activity	93.51 (19.48)	98.57 (9.42)	−5.06 (−6.97, 3.16)	**<0.001**
Pain/discomfort	88.67 (22.88)	96.35 (13.32)	−7.67 (−9.93, 5.42)	**<0.001**
Anxiety/depression	94.20 (18.81)	98.64 (8.44)	−4.43 (−6.27, 2.59)	**<0.001**
Utility index	0.9212 (0.1701)	0.9787 (0.0811)	−0.0575(−0.0673, 0.0477)	**<0.001**

The results of multivariate linear regression for the EQ-5D index are shown in Table 3. Hypertensive patients with lower education attainment (P = 0.045), complications (P < 0.001), lower family income (P = 0.004), and a family history of HBP (P = 0.005) reported lower HRQoL than did those without complications. Among the normotensive individuals, those who were older (P = 0.001), spent higher annual health expenditure (P < 0.001), had lower education attainment (P < 0.001), had lower household income (P < 0.001), lived in rural areas (P = 0.008), and had a family history of HBP (P = 0.012) tended to report lower HRQoL.

**Table 3:** Multivariate linear regression of hypertension group and normal population in Shandong, China, 2014.

Variables	Hypertensives			Normotensives		
	β	SE.	P	β	SE.	P
Age			0.847			**0.001**
<40 (ref.)						
40-	-0.036	0.043	0.405	0.001	0.004	0.750
50-	-0.026	0.040	0.526	−0.012	0.004	**0.002**
Education			**0.045**			**<0.001**
Primary or below (ref.)						
Junior	0.043	0.020	**0.030**	0.013	0.004	**<0.001**
Senior or above	−0.009	0.029	0.754	0.019	0.005	**<0.001**
Occupation				—		
Others (ref.)						
Farmers	0.004	0.022	0.865	-0.006	0.004	0.083
Household income			**0.004**			**<0.001**
<20000- (ref.)						
20000-	0.045	0.021	**0.028**	0.012	0.004	**0.002**
40000-	0.061	0.023	**0.007**	0.019	0.004	**<0.001**
Sleeping time			0.806			0.077
<7 (ref.)						
7-	0.013	0.018	0.467	0.002	0.003	0.535
8-	−0.006	0.022	0.783	0.008	0.004	0.069
Health expenditure			0.083			**<0.001**
<900 (ref.)						
900-	0.017	0.022	0.783	−0.005	0.004	0.141
1800-	−0.031	0.019	0.110	−0.018	0.003	**<0.001**
Residence place						
Urban (ref.)						
Rural	−0.028	0.027	0.306	0.013	0.005	**0.008**
Family history of HBP						
No (ref.)						
Yes	−0.047	0.017	**0.005**	−0.010	0.004	**0.012**
Complications				—	—	—
No (ref.)						
Yes	-0.060	0.017	**<0.001**			
Antihypertensive medication*				—	—	—
No (ref.)						
Yes	−0.016	0.018	0.389			
Willingness to acquire health-related knowledge						
No (ref.)						
Yes	0.029	0.019	0.123	0.005	0.003	0.109
HBP knowledge level			0.137			0.390
0 (ref.)						
1	0.055	0.032	0.088	0.000	0.006	0.943
2	0.014	0.029	0.626	0.004	0.005	0.408
3	0.041	0.024	0.080	0.003	0.004	0.434
Constant	0.941	0.080	**<0.001**	0.957	0.013	**<0.001**

Note: Dependent variable: EQ-5D utility index; ref.: reference group.

*refers to antihypertensive medication in the past two weeks.

## Discussion

Our research showed that hypertensive patients reported significantly lower scores in each of the EQ-5D domains and a lower health utility index than did normotensive individuals, suggesting that hypertension is associated with HRQoL^15,16^. Similar to a previous study, we found that the score in the domain of pain/discomfort among the hypertensive individuals was significantly lower than that among the normotensive individuals. Some clinical studies have shown a relationship between disease severity and body pain/discomfort^15^. Hypertensive individuals with body pain/discomfort might have a poorer HRQoL than the general population. This study indicated that hypertensive individuals had poorer scores in anxiety/depression than normotensive individuals did. Saboya P *et al*. found that anxiety and, especially, depressive symptoms were significantly associated with hypertension and had an adverse effect on hypertensive patients’ quality of life^20^, which was consistent with our study. Some studies found lower physical capacity among hypertensive individuals compared with normotensive individuals^15^, which might explain the findings in the current study that hypertension was related to lower scores in mobility, self-care and usual activity.

The current study showed that family history of HBP was an important risk factor in HRQoL for both hypertensive and normotensive individuals^21^. A previous study indicated that family history of HBP might exert stress on family members, which could result in anxiety, depression, and other mental disorders. Such psychological disorders would adversely impact the HRQoL of the family members with a history of HBP^22^. In addition, lower household income was found to be associated with poorer HRQoL for both hypertensive and normotensive individuals. People with low household income tended to have higher healthcare needs but lacked the financial capacity to pay for health services. The discomfort caused by the lack of healthcare utilization exaggerates the dissatisfaction of self-rated health, which inevitably has a negative effect on people’s HRQoL^23^.

Complications with other diseases are relatively common in hypertensive patients, such as angina pectoris, myocardial infarction, and stroke^15^. Our research observed that hypertensive patients with such complications (angina pectoris, myocardial infarction, stroke, and hypertensive nephropathy) tended to have lower HRQoL compared to those without such complications. There are two explanations for this result. First, the complications of hypertension might contribute to poor ADL (e.g., stroke) and bodily pain (e.g., angina pectoris), with the result that lower HRQoL among these patients might be due to subsequent complications, not to the hypertension itself^24–26^. Second, the complications might affect hypertensive patients’ perception of their general health condition^27^, which influences how the patients rate their HRQoL. Moreover, other complications^28^ (e.g., nephropathy, retinopathy) that we did not include in this study may have had an effect on HRQoL in the hypertensive individuals.

In addition, we found that HRQoL in the normotensive individuals was associated with factors including age, educational level, and health expenditure. High health expenditure was considered an economic burden to the household, and it often placed households at higher risk of catastrophic health expenditure and pushed them into poverty. People with higher educational levels tended to have higher levels of health literacy, which was considered helpful for improving HRQoL^29^. Further, living in rural areas and being older were found to be risk factors of HRQoL in our study, findings that have been previously verified^22,30^.

The EQ-5D used in this study is a simple, well-documented, and validated generic instrument^31^. It has been widely used in China to measure HRQoL in recent years^13,30^. Similar to other instruments measuring HRQoL, the EQ-5D can reflect the health utility index score. However, the utility index score cannot be generated by the direct measures of the EQ-5D. A population-based time trade-off (TTO) model can help to convert the measurement results of the EQ-5D into health utility index scores^21^. In this study, both the direct measurements and the health utility index scores of the EQ-5D were analysed, which can provide real profiles of HRQoL among hypertensive and normotensive individuals. This is the first study in China to compare HRQoL between hypertensive and normotensive individuals using the EQ-5D.

This study has a number of strengths. First, this study is one of the only studies to measure the EQ-5D by health utility index scores based on a Chinese time TTO model, which provides a real profile of EQ-5D utility index scores among hypertensive individuals in China. Second, this is the first study in China to compare HRQoL between hypertensive and normotensive individuals using the EQ-5D.

The present study also has certain limitations. First, information including willingness to acquire health-related knowledge and the EQ-5D were self-reported, leading to the possibility of subjective bias. Second, as a cross-sectional design, the correlation between HRQoL and associated factors cannot be interpreted as causal. Third, there might be other potential factors that affect HRQoL, such as medication adherence, hospital admission, and other chronic diseases, which might cause a deviation of the results and will be remedied in the follow-up study.

## Conclusion

This population-based study found that individuals with hypertension had lower HRQoL compared with those without hypertension. Complications in the individuals with hypertension were found to be associated with even lower HRQoL. Educational level, household income and family history of HBP were found to be important risk factors of HRQoL in both hypertensive and normotensive individuals, which indicates that existing health insurance schemes may need to be modified to improve their benefit to poor individuals. This study also identified a number of associated factors for HRQoL among normotensive individuals, including age, residence, and health expenditure, providing potential evidence for policy design (e.g., to promote health literacy among those with poor education) to improve HRQoL among normotensive individuals.
